# A sequence-based approach to identify reference genes for gene expression analysis

**DOI:** 10.1186/1755-8794-3-32

**Published:** 2010-08-03

**Authors:** Raj Chari, Kim M Lonergan, Larissa A Pikor, Bradley P Coe, Chang Qi Zhu, Timothy HW Chan, Calum E MacAulay, Ming-Sound Tsao, Stephen Lam, Raymond T Ng, Wan L Lam

**Affiliations:** 1 Department of Integrative Oncology, British Columbia Cancer Agency Research Centre, Vancouver, BC, Canada; 2 Ontario Cancer Institute/Princess Margaret Hospital, Toronto, ON, Canada; 3 Department of Computer Science, University of British Columbia, Vancouver, BC, Canada

## Abstract

**Background:**

An important consideration when analyzing both microarray and quantitative PCR expression data is the selection of appropriate genes as endogenous controls or reference genes. This step is especially critical when identifying genes differentially expressed between datasets. Moreover, reference genes suitable in one context (e.g. lung cancer) may not be suitable in another (e.g. breast cancer). Currently, the main approach to identify reference genes involves the mining of expression microarray data for highly expressed and relatively constant transcripts across a sample set. A caveat here is the requirement for transcript normalization prior to analysis, and measurements obtained are relative, not absolute. Alternatively, as sequencing-based technologies provide digital quantitative output, absolute quantification ensues, and reference gene identification becomes more accurate.

**Methods:**

Serial analysis of gene expression (SAGE) profiles of non-malignant and malignant lung samples were compared using a permutation test to identify the most stably expressed genes across all samples. Subsequently, the specificity of the reference genes was evaluated across multiple tissue types, their constancy of expression was assessed using quantitative RT-PCR (qPCR), and their impact on differential expression analysis of microarray data was evaluated.

**Results:**

We show that (i) conventional references genes such as *ACTB *and *GAPDH *are highly variable between cancerous and non-cancerous samples, (ii) reference genes identified for lung cancer do not perform well for other cancer types (breast and brain), (iii) reference genes identified through SAGE show low variability using qPCR in a different cohort of samples, and (iv) normalization of a lung cancer gene expression microarray dataset with or without our reference genes, yields different results for differential gene expression and subsequent analyses. Specifically, key established pathways in lung cancer exhibit higher statistical significance using a dataset normalized with our reference genes relative to normalization without using our reference genes.

**Conclusions:**

Our analyses found *NDUFA1*, *RPL19*, *RAB5C*, and *RPS18 *to occupy the top ranking positions among 15 suitable reference genes optimal for normalization of lung tissue expression data. Significantly, the approach used in this study can be applied to data generated using new generation sequencing platforms for the identification of reference genes optimal within diverse contexts.

## Background

Gene expression profiling, including quantitative RT-PCR (qPCR) and microarray experimentation, is invaluable for the molecular analysis of biological systems. The interpretation of results from such experiments (i.e., the determination of differential expression for a particular gene among datasets) is strongly influenced by the selection of reference genes for normalization across datasets [1]. Specifically, gene expression is normalized within a given dataset by calculating the transcript abundance of the gene of interest relative to a gene that is constantly expressed across independent datasets (termed a "housekeeping" or a "reference" gene), and differential expression between two datasets or samples is determined by calculating the ratio of the normalized expression levels for the gene of interest between the two datasets. Typically, housekeeping genes satisfy the following criteria: they are highly expressed in the cell, the variability in expression between samples is minimal, and the genes' expression is not influenced by the experimental conditions tested [2]. Hence, problems arise when housekeeping genes are selected that do not meet these criteria, as fluctuations in these genes may erroneously influence the data interpretation.

Historically, *beta actin *(*ACTB*), *glyceraldehyde-3-phosphate dehydrogenase *(*GAPDH*), and *18 S rRNA *have been routinely used as reference genes for qPCR and microarray data normalization. However, a number of studies have shown that expression of these genes varies considerably depending on the specific tissue type and disease state of the tissue [3-16]. Attempts to achieve more reliable normalization include the spiking of synthetic poly-A RNAs for the analysis of cDNA arrays and northern blots, and the combined use of an oligo-(dT)n primer with an 18 S specific primer for qPCR analysis [17,18]. In addition, re-mining of large microarray datasets for the identification of novel, highly stable genes, as well as use of a combination of reference genes instead of a single gene for normalization, are some of the other approaches taken to address this problem [11,13,19].

Recently, efforts have been made to identify more suitable reference genes for microarray and qPCR studies of lung cancer. Specifically, candidate reference genes have been identified from the mining of microarray gene expression data to identify the least variable genes, followed by validation of expression using qPCR [11,20,21]. However, as microarray data do not provide absolute abundance values for transcripts, selection of reference genes from this type of data is inherently problematic. To circumvent this handicap in the utilization of microarray data, we turn to the use of large-scale expression profiling permitted by serial analysis of gene expression (SAGE) experimentation for the identification of novel reference genes optimal for the study of lung cancer. This approach, which we have termed normalization of expression by permutation of SAGE (*NEPS*), takes advantage of the fact that SAGE is a transcriptome profiling technique that identifies the absolute abundance levels of transcripts by direct enumeration of sequence tag counts, thus allowing the direct comparison of expression levels across multiple profiles without the need for reference or housekeeping genes [22].

*NEPS *adopts a permutation test approach designed for analyzing relatively small sample sizes, such as those typically encountered with SAGE. Unlike the conventional T-test, the permutation test is non-parametric [23]. The null hypothesis states that the mean gene expression levels in two groups of SAGE libraries being compared (in this case normal and cancer), are the same. For this analysis, samples from both the normal and the cancer groups are pooled, followed by random sampling to create a simulated Group 1 and a simulated Group 2. For each gene, the difference in expression between these two simulated groups was measured. This exercise was repeated 10,000 times, thus generating a simulated mean μ and a simulated standard deviation *σ*. The *permutation score *(PS) of a given gene is defined by , where *O *is the true difference between the average expression levels in the two groups. Hence, for a given gene, the closer the permutation score is to zero, the more it satisfies the constancy requirement.

To demonstrate the utility of *NEPS *for selecting genes that satisfy the constancy requirement, we analyzed 24 bronchial epithelial lung SAGE libraries, 2 lung parenchyma libraries, and 11 lung squamous cell carcinoma libraries. From this analysis, *NEPS *selected 15 genes, which we hereafter refer to as the lung-*NEPS *reference genes (Table 1). We further demonstrate that (1) while these genes perform well as reference genes for lung, they are not satisfactory for normalization of expression data from other tissues, suggesting that reference genes are tissue-specific, and (2) in lung cancer datasets, differential gene expression determination and subsequent pathway analyses are improved after normalization using the lung-*NEPS *reference genes.

**Table 1:** Lung *NEPS *Genes

*Gene Symbol*	*Gene Name*	***Average Raw Tag Count***^***1***^	*Permutation Score*
*PPP1CB*	*protein phosphatase 1, catalytic subunit, beta isoform*	29	0.003
*B2M*	*beta-2-microglobulin*	829	0.011
*CSTB*	*cystatin B (stefin B)*	52	0.036
*RPL4*	*ribosomal protein L4*	46	0.045
*SLFN13*	*schlafen family member 13*	31	0.045
*CAPZB*	*capping protein (actin filament) muscle Z-line, beta*	77	0.050
*ATP5J*	*ATP synthase, H+ transporting, mitochondrial F0 complex, subunit F6*	38	0.059
*RAB5C*	*RAB5C, member RAS oncogene family*	44	0.064
*NDUFA1*	*NADH dehydrogenase (ubiquinone) 1 alpha subcomplex, 1, 7.5 kDa*	89	0.077
*RPL19*	*ribosomal protein L19*	69	0.082
*HMGB1*	*high-mobility group box 1*	39	0.087
*CD55*	*CD55 molecule, decay accelerating factor for complement (Cromer blood group)*	27	0.100
*RPS18*	*ribosomal protein S18*	112	0.123
*HSPA1A*	*heat shock 70 kDa protein 1A*	40	0.133
*EIF4A2*	*eukaryotic translation initiation factor 4A, isoform 2*	89	0.145

^1^Across all normal and cancer SAGE libraries

## Methods

### SAGE library construction

26 normal and 11 lung cancer SAGE libraries were constructed and used in the analysis [24]. The construction of the 26 normal libraries has been previously described [25,26]. 24 of these libraries were generated from exfoliated bronchial cells obtained from bronchial brushes, and two libraries from normal lung parenchyma (Additional file 1). Conversely, the 11 cancer libraries were generated from biopsied specimens with six libraries representing lung squamous cell carcinoma and five libraries representing carcinoma in situ. This data can be found at the GEO database with the following series accession numbers: GSE3707, GSE5473, and GSE7898. All samples were acquired under approval by the University of British Columbia - British Columbia Cancer Agency Research Ethics Board (UBC-BCCA-REB) and all subjects provided written consent.

### SAGE data from public domain

Publicly available SAGE data were also used in this analysis, representing both brain and breast cancer. Specifically, six normal and 12 breast cancer libraries (Additional file 2) and 7 normal and 19 brain cancer libraries were used (Additional file 3). The libraries were obtained from the cancer genome anatomy project (CGAP) database http://cgap.nci.nih.gov[27,28].

### Permutation test

Given that SAGE libraries are expensive to generate, the number of libraries in a given study is typically small (i.e., in 10's, rather than in 100's). The permutation test is a non-parametric test, which does not assume any underlying distribution. The number of samples required for the test to achieve sufficient statistical power is relatively low compared to other statistical tests (e.g., t-test and χ^2^-square test). Furthermore, each additional sample increases the power of the test exponentially. The permutation test is a test of the means between two different distributions. Without loss of generality, let us assume that one distribution is for the gene expression level of a particular gene in normal tissues (i.e. subscript *n*), and that the other distribution is for cancerous tissues (i.e. subscript *c*). Genes are selected using the following hypotheses:

If there is little difference between the two means, it would make no difference if we mix the cancerous samples with the normal samples. But, if the null hypothesis is rejected, it indicates that the gene expression levels of normal and cancer samples are sufficiently different (the alternative hypothesis). In the following, we show our specific implementation of the test. Let *n *and *c *be the number of normal tissue samples and the number of cancerous tissue samples respectively.

A. For each gene, select all the gene-specific normalized tag counts from the normal libraries and all the gene-specific normalized tag counts from the cancer libraries.

B. Randomly select *n *counts to create a *simulated *normal set, and calculate the simulated normal mean *μ*_*sn*_.

C. Similarly, select the remaining *c *counts form the simulated cancerous set. Calculate the simulated cancer mean *μ*_*sc*_.

D. Consider the random variable *v *= *μ*_*sc *_- *μ*_*sn*_, called the *simulated difference*.

E. Repeat the steps A to D above *m *times. Let *μ *and *σ *denote the mean and the standard deviation of *v*.

F. Now separate the libraries back into their true identity: normal or cancerous. Calculate the true observed difference *O *= *μ*_*rc *_- *μ*_*rn*_, where *μ*_*rc *_denotes the true mean count of the cancerous libraries, and *μ*_*rn *_denotes the true mean of the normal libraries.

G. Calculate the Permutation Score *PS *where .

H. Repeat all the above steps for each gene. Sort the permutation score in descending order.

The permutation score is one way to measure how likely the actual observed difference occurs by chance. It is based on standardization, i.e., subtracting the mean and then divided by the standard deviation. The more the true observed difference is from the average (expressed as multiples of the standard deviation), the less likely that the true observed difference is a coincidence. That is to say, the larger the permutation score, the more significant is the observed difference between cancerous and normal samples.

On the other hand, for the sake of evaluating the constancy requirement, the ideal reference gene would have a permutation score equal to 0. This means that there is no difference in the distributions of expression levels between cancerous and normal samples. For the results reported here, we used *m = 10,000 *permutations.

### Data pre-processing

Raw tag counts for each SAGE library were normalized to tags per million (TPM) to facilitate adequate comparison among libraries. Tag-to-gene mapping was performed using the February 5^th^, 2007 version of *SAGEGenie *[27]. In cases where multiple SAGE tags mapped to the same gene, the tags were collapsed to capture all potential transcript variants, and a cumulative tag count was utilized for analysis.

### Statistical criteria for reference gene selection

The permutation test outlined above was used to identify genes which were statistically similar when comparing the libraries from normal tissue (bronchial epithelium and lung parenchyma) and cancerous tissue of the lung. Three main criteria were used for reference gene selection: permutation score (described above) ≤ 0.15; at least two SAGE tags observed in each library; and an overall average count of ≥ 25 across all samples. For the analysis in brain and breast tissue, the first two criteria were maintained, but due to the lower sequencing depth, an average count of ≥ 10 across all samples was used instead.

### Quantitative RT-PCR validation in clinical lung cancer specimens

One microgram of total RNA from 15 lung tumor and matched non-malignant parenchyma samples were converted to cDNA using the High-Capacity cDNA archive kit (Applied Biosystems Inc., Foster City CA). One hundred nanograms of cDNA were utilized for qPCR using the TaqMan Gene Expression Assay (Applied Biosystems Inc). All fifteen lung NEPS genes and six additional reference genes were assayed. All TaqMan probes were pre-optimized by Applied Biosystems. Primer IDs for all genes are provided in Additional file 4. The 30 samples were assayed in triplicate in parallel along with negative (no cDNA template) controls using the 7500 Fast Real-Time PCR System. Appropriate cDNA dilutions were used such that the exponential phase of the amplification curves were within the 40 PCR cycles recommended by the manufacturer (i.e. ranging from 16-36 cycles for the 20 genes and 1-13 cycles for 18SRNA). Cycle thresholds were determined from amplification curves using 7500 Fast System software.

For the analysis of qPCR data, three different methods were used. Within each method, all genes were ranked from best to worst. Subsequently, for each gene, a cumulative ranking across all three methods was determined by summing its rank from each individual method. Two previously published methods, *geNorm *[14] and *NormFinder *[29], and the variance of cycle threshold difference (dCt) across all 15 tumor/matched non-malignant sample pairs were the approaches used to determine constancy.

### Analysis of publicly available microarray datasets

Lung NEPS genes were used to re-normalize two publicly available microarray datasets. Microarray data were obtained from *GEO *at NCBI under accession numbers GSE10072 [30] and GSE12428 [31].

For the Affymetrix data (GSE10072), Raw CEL files were processed through Affymetrix's Microarray Array Suite (MAS) 5.0 algorithm in the "affy" package in *Bioconductor *[32,33]. Briefly, MAS 5.0 is a three step process which involves a global background signal correction, correction of the probe value for cross-hybridization and spurious signals using mismatch probes which are off by one base, and finally, scale normalization of each experiment to a fixed median intensity to facilitate inter-experimental comparison http://media.affymetrix.com/support/technical/whitepapers/sadd_whitepaper.pdf. Probes were filtered on MAS 5.0 calls, and those having a "P" or "M" call in at least 50% of samples were retained. This resulted in a dataset of 11440 probes. Of the 15 lung NEPS reference genes, 12 were represented on the array platform. Of those 12 genes, probes which had a "P" call in 100% of the samples were used for the calculation of the scaling factor with only one probe/gene allowed. If two probes met these criteria for one gene, the probe with the highest mean expression was chosen. After employing these criteria, eight probes were used (Additional file 5), which represented genes *PPP1CB*, *B2M*, *RPL4*, *CAPZB*, *ATP5J*, *RAB5C*, *NDUFA1*, and *HSPA1A*.

For the Agilent microarray data (GSE12428), all lung NEPS genes were represented on this microarray platform. Data was processed as described previously [31]. In the cases where lung NEPS genes were represented with multiple probes, the probe with the maximum average intensity across the dataset was used. A list of the probes used is given in Additional file 6. Since each sample had at least two replicate experiments, the average across replicate experiments was used for each probe.

To determine the scaling factor, for each sample, linear regression analysis was performed comparing the values for the reference gene (x) versus the average values for the reference genes across the sample set (y). The slope of the line based on least-squares fitting was then multiplied to each value in the experiment.

Next, Significance Analysis of Microarrays (SAM) was performed to determine differentially expressed genes between non-malignant and malignant samples for both microarray datasets using the "samr" package in *R *[34]. Unpaired analysis was performed using the normal samples versus tumor samples and the delta parameter set to 0.4. Probes which had a Q-value% ≤ 5 were considered significant. For the Affymetrix dataset, results were compared between the dataset normalized with *MAS 5.0 *alone and *MAS 5.0 *+ NEPS scaling and for the Agilent dataset, the comparison was done between median normalization alone and NEPS scaling followed by median normalization.

## Results and Discussion

### Identification of reference genes for gene expression analysis in lung cancer

From our *NEPS *analysis [with an imposed permutation score (PS) threshold ≤ 0.15, and an average expression of ≥ 25 raw tag counts across all samples], 15 genes were identified as the most consistently expressed across normal and cancerous lung tissue (Table 1). Here we identified *beta-2-microglobulin *(*B2M*), components of the large ribosomal subunit such as *ribosomal protein L19 *(*RPL19*) and *ribosomal protein L4 *(*RPL4*), components of the small ribosomal subunit such as *ribosomal protein S18 *(*RPS18*), and electron transport chain constituents such as *NADH dehydrogenase (ubiquinone) 1 alpha subcomplex 1 *(*NDUFA1*), to rate highly in our permutation analysis, thereby suggesting their potential as reliable reference genes. *B2 M *has previously been utilized as a reference gene [10,15], providing validity to the approach used here. The 18 S and 28 S rRNAs have previously served as reference genes [4,7,11,12,16], and here we show that the ribosomal protein genes can also provide this service.

### Performance of standard and previously reported reference genes

**Figure 1:**
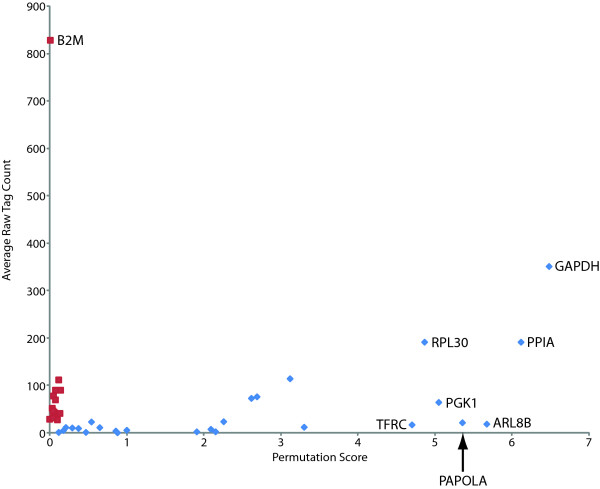
**Enhanced performance of the lung-*NEPS *genes (red; see Table 1) relative to previously reported and standard reference genes traditionally used in qPCR and microarray normalization (blue)**. The x-axis represents the permutation score of a defined gene and the y-axis represents the average raw (non-normalized) tag count for the same gene. Data used in the graph are given in Additional file 7. Lung NEPS genes are stable and highly expressed as compared to the traditionally used genes. *B2 M *appears to perform the best with respect to high average tag count and low permutation score. Notably, the gene that performs the poorest is *GAPDH*.

A previous study reported a meta-analysis of microarray data designed to identify novel reference genes for the study of non-small cell lung cancer (NSCLC) [11]. Using a small panel of tumor/normal specimens, the authors demonstrated that genes commonly used for reference in qPCR experimentation were sub-optimal, and identified novel, more consistently expressed genes to be superior as reference genes. Additionally, studies of asthmatic airways have also shown that traditional reference genes such as *ACTB *and *GAPDH *perform poorly in this regard [6,7,12,14,15]. We find similar results using our *NEPS*-based approach. As described above, genes such as *B2 M *(permutation score = 0.011) and *RPL19 *(0.082) were shown to have very low permutation scores denoting stable expression between normal and cancer, whereas *ACTB *(2.69) and *GAPDH *(6.48) performed very poorly with significant differential expression. Other known reference genes such as *hypoxanthine phosphoribosyltransferase 1 *(*HPRT1*) (0.114) and *TATA box binding protein *(*TBP*) (0.468), while exhibiting low permutation scores, were not as highly expressed with average raw tag counts across all samples of 1.54 and 1.65, respectively. In contrast, genes such as *peptidylprolyl isomerase A (PPIA, aka cyclophilin A) *(6.11), *transferrin receptor (TFRC, p90, CD71) *(4.70), and *phosphoglycerate kinase 1 *(*PGK1*) (5.04) identified in a microarray meta-analysis study (see above) [11], performed poorly in our study, as revealed by the relatively high permutation scores. Although these particular genes did not perform as well as the reference genes identified from our permutation analysis, other genes identified by Saviozzi *et al*., such as *signal transducer and activator of transcription 1 *(*STAT1*) (0.21), *esterase D/formylglutathione hydrolase *(*ESD*) (0.18), *Yes-associated protein 1 *(*YAP1*) (0.65) and *polymerase (RNA) II (DNA directed) polypeptide A *(*POLR2A*) (0.88) did perform satisfactorily in our study, as evidenced by permutation scores ≤ 1. In addition to these genes, a second set of genes identified using a cross-tissue and cross-platform analysis were also assessed [20] and similarly, while some genes such as *C-terminal binding protein *1 (*CTBP1*), *cullin 1 *(*CUL1*), *DIM1 dimethyladenosine transferase 1-like *(*DIMT1L*), *tripartite motif-containing *27 (*TRIM27*) and *ubiquilin 1 *(*UBQLN1*) performed reasonably well based on our metric, others such as *poly(A) polymerase alpha *(*PAPOLA*) and *ADP-ribosylation factor-like 8B *(*ARL8B*) did not (Figure 1, Additional file 7).

### Demonstrating tissue specificity of reference genes

**Figure 2:**
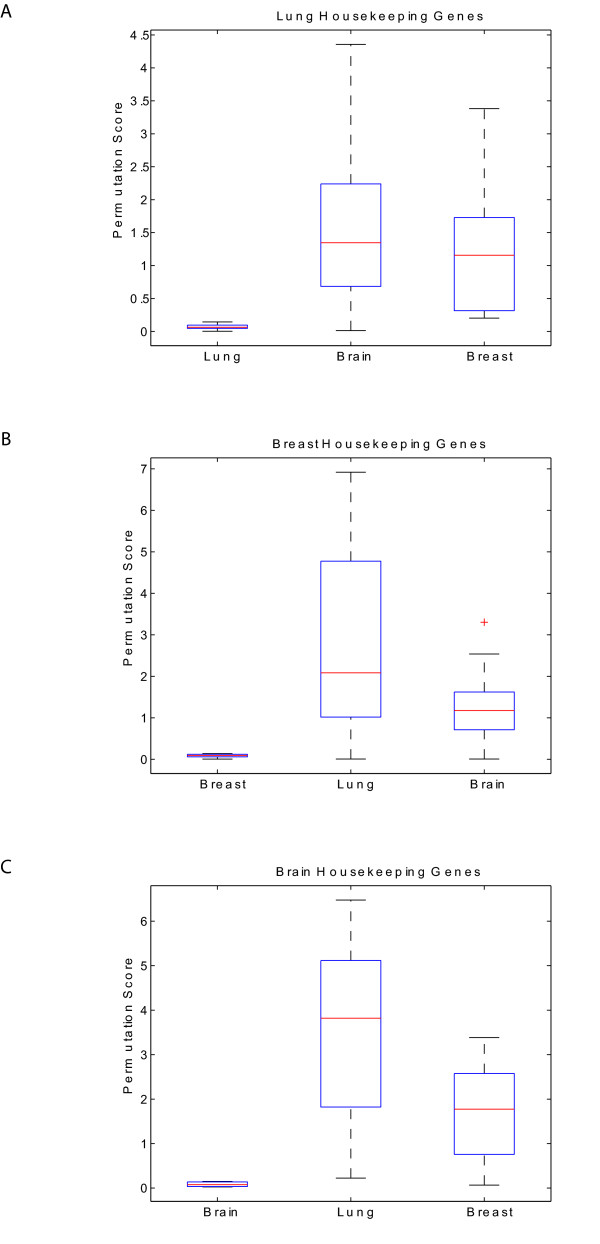
**Tissue-specificity of reference genes**. Comparison of the permutation scores for reference genes generated in one tissue type with permutation scores for the same genes in the other two tissue types. (A) Performance of lung-*NEPS *genes in breast and brain tissues, (B) Performance of breast-*NEPS *genes in lung and brain tissues, and (C) Performance of brain-*NEPS *genes in lung and breast tissues.

To further our investigations regarding reference genes optimal for cancer cell biology, we expanded our analysis to include publicly available SAGE libraries representing normal and cancer tissue from both brain and breast. The results of this analysis clearly demonstrate that the reference genes identified in the lung dataset are distinct from those found in either breast (Table 2) or brain (Table 3). This data strongly suggests that reference genes should be selected in a tissue specific manner. For example, *GAPDH*, which performed poorly as a reference gene for lung gene expression analysis (see above), was in fact one of the best reference genes identified from the analysis of the brain dataset (Table 3). Moreover, not only was there no overlap among the reference gene lists determined for each of the three different tissue types (i.e., lung-*NEPS*, breast-*NEPS*, brain-*NEPS*), but when examining reference genes specific to one tissue type (i.e. lung-*NEPS*) in the other two tissue types (i.e. breast or brain), the permutation scores for these genes were significantly higher and more variable (Figure 2). These results are consistent with other studies demonstrating the need for tissue and context-specific selection of reference genes [3,5,8,10,14].

**Table 2:** Breast *NEPS *Genes

*Gene Symbol*	*Gene Name*	*Average Raw Tag Count*	*Permutation Score*
*EIF5A*	eukaryotic translation initiation factor 5A	22	0.003
*EIF3S2*	eukaryotic translation initiation factor 3, subunit 2 beta, 36 kDa	12	0.037
*RPS8*	ribosomal protein S8	122	0.046
*TSPAN9*	tetraspanin 9	122	0.051
*UBB*	ubiquitin B	39	0.057
*RPL28*	ribosomal protein L28	78	0.064
*FTL*	ferritin, light polypeptide	16	0.066
*YWHAQ*	tyrosine 3-monooxygenase/tryptophan 5-monooxygenase activation protein, theta polypeptide	19	0.074
*TMEM49*	transmembrane protein 49	13	0.083
*FAM39B*	family with sequence similarity 39, member B	11	0.091
*NINJ1*	ninjurin 1	13	0.097
*RPL30*	ribosomal protein L30	108	0.108
*PDE6B*	phosphodiesterase 6B, cGMP-specific, rod, beta	10	0.115
*TUBA3*	tubulin, alpha 1a	50	0.117
*MYL9*	myosin, light chain 9, regulatory	15	0.120
*MYH9*	myosin, heavy chain 9, non-muscle	21	0.128
*NPM1*	nucleophosmin (nucleolar phosphoprotein B23, numatrin)	48	0.130
*HLA-A*	major histocompatibility complex, class I, A	45	0.131
*RPS2*	ribosomal protein S2	63	0.138

**Table 3:** Brain *NEPS *Genes

*Gene Symbol*	*Gene Name*	*Average Raw Tag Count*	*Permutation Score*
*NUCKS1*	*nuclear casein kinase and cyclin-dependent kinase substrate 1*	14	0.024
*CDAN1*	*congenital dyserythropoietic anemia, type I*	35	0.030
*PABPCP2*	*poly(A) binding protein, cytoplasmic, pseudogene 2*	15	0.033
*GTF2I*	*general transcription factor II, i*	22	0.036
*ZFAND5*	*zinc finger, AN1-type domain 5*	20	0.060
*GAPDH*	*glyceraldehyde-3-phosphate dehydrogenase*	163	0.068
*NCL*	*nucleolin*	13	0.083
*FIS1*	*fission 1 (mitochondrial outer membrane) homolog (S. cerevisiae)*	10	0.094
*GRIN2C*	*glutamate receptor, ionotropic, N-methyl D-aspartate 2C*	81	0.132
*RPS27A*	*ribosomal protein S27a*	63	0.142
*COX4I1*	*cytochrome c oxidase subunit IV isoform 1*	17	0.148
*CXXC5*	*CXXC finger 5*	13	0.149

### Quantitative RT-PCR validation of identified reference genes in lung cancer samples

Using a secondary set of 15 tumor and matched non-malignant samples, qPCR was used to validate consistency of expression for all lung-*NEPS *genes. Additionally, we performed qPCR for previously identified, commonly used housekeeping genes *ACTB*, *GAPDH*, *HPRT1*, and *TBP*. In addition, two genes out of 13 identified (*CUL1 *and *TRIM27*) as suitable reference genes from a previously published study [20], were selected here based on high *NEPS *performance (see above), for qPCR analysis.

Of the *NEPS *genes analyzed, *NDUFA1*, *RPL19*, *RAB5C, member RAS oncogene family *(*RAB5C*), and *RPS18 *performed the best based on the cumulative ranking metric (Table 4). Conversely, the standard reference genes *ACTB*, *GAPDH*, and *HPRT1 *did not perform as well. These results confirm a high constancy of expression for a subset of the lung-*NEPS *genes using an alternative method in a secondary set of samples.

**Table 4:** Quantitative RT-PCR analysis of lung NEPS genes and select previously identified genes

Gene Symbol*	*Cumulative Rank*	*dCt Variance*	*Rank*	*NormFinder Stability Value*	*Rank*	*geNorm* *M value*	*Rank*
***NDUFA1***	11	2.011	4	0.059	2	1.141	5
***RPL19***	14	2.252	6	0.071	4	1.140	4
***RAB5C***	18	2.928	10	0.058	1	1.150	7
***RPS18***	20	0.011	1	0.064	3	1.461	16
*TBP*	24	3.846	16	0.076	7	1.097	1
***RPL4***	27	1.523	3	0.099	12	1.253	12
***ATP5J***	28	2.150	5	0.090	9	1.342	14
***HMGB1***	29	2.648	8	0.093	10	1.210	11
*TRIM27*	29	3.752	15	0.073	5	1.158	9
***EIF4A2***	31	3.229	12	0.106	16	1.131	3
***CAPZB***	33	4.362	18	0.100	13	1.105	2
***PPP1CB***	33	3.453	13	0.104	14	1.143	6
*CUL1*	34	5.164	20	0.075	6	1.156	8
*ACTB*	37	2.731	9	0.099	11	1.638	17
***B2M***	38	1.517	2	0.154	21	1.369	15
*HPRT1*	39	3.611	14	0.105	15	1.173	10
***CSTB***	41	3.026	11	0.108	17	1.259	13
***CD55***	44	2.460	7	0.131	18	1.811	19
***HSPA1A***	47	7.330	21	0.077	8	1.653	18
*GAPDH*	58	4.044	17	0.145	20	2.093	21
***SLFN13***	58	4.803	19	0.132	19	1.858	20

*Genes identified in this study are bolded

### Effect of reference genes on differential gene expression analysis

**Figure 3:**
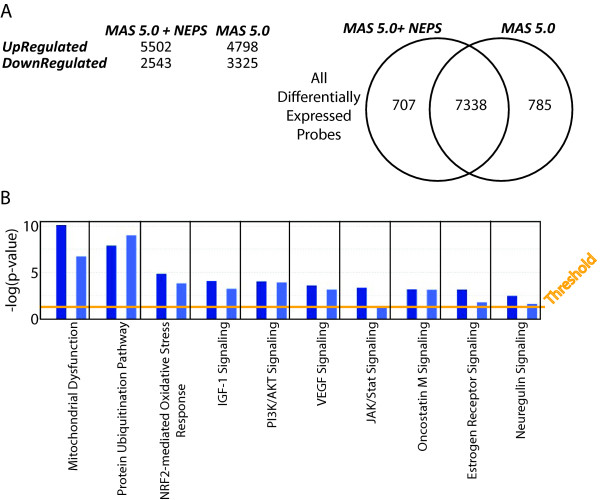
***SAM *and pathway analysis of a dataset normalized with and without lung NEPS genes**. (A) Number of probes identified as differentially over and underexpressed between cancer and normal using *SAM *on the dataset with and without NEPS normalization. Venn diagram illustrates the overlap in the genes identified as well as those which are different between the two analyses. (B) Canonical pathway analysis using *Ingenuity Pathway Analysis*. Dark blue bars represent the results from the dataset normalized with *MAS 5.0 *+ *NEPS *and light blue bars represent the results from normalization using *MAS 5.0 *alone. The pathways which are the most significant are the most significant in both analyses. Note that key pathways such as *Neuregulin *signaling and *JAK/Stat *are identified with higher significance when normalized using the lung NEPS genes. Such differences illustrate the impact of reference gene selection and normalization on differential gene expression analysis.

Using a publicly available microarray dataset (GSE10072, [30]), differential expression analysis was performed using *SAM *[34]. Results from *SAM *were compared using the dataset normalized by *MAS 5.0 *alone, versus the same dataset normalized by *MAS 5.0 *with scale normalization using the lung-*NEPS *reference genes represented on the microarray. We observed differences in the total number of differentially regulated genes, depending on the normalization protocol used. When *MAS 5.0 *+ *NEPS *normalization was used, 5502 genes were identified as up-regulated in cancer, whereas 4798 up-regulated genes were identified using *MAS 5.0 *alone. With respect to down-regulated genes, 2543 were identified using *MAS 5.0 *+ *NEPS*, whereas 3325 were identified using *MAS 5.0 *alone (Figure 3A). According to the *Canonical Pathway Analysis *[*Ingenuity Pathway Analysis *(IPA)], we observe slight differences in both the number and the significance of identified pathways between the two sets of differentially normalized microarray data (Additional file 8). For example, while both datasets identify pathways such as mitochondrial dysfunction and protein ubiquitination, analysis of the dataset normalized by *MAS 5.0 *+ *NEPS *identifies pathways known to be important in lung cancer, such as *Neuregulin *and *JAK/Stat *[35], at a higher significance relative to analysis of the same dataset normalized by *MAS 5.0 *alone (Figure 3B). Similarly, when evaluated using an additional publicly available lung cancer microarray dataset [31], we observe slight differences between the various pathways identified from analysis of differentially expressed genes derived from a *NEPS*-normalized dataset versus a dataset not normalized using the lung-*NEPS *genes (Additional file 9, Additional file 10). These results demonstrate that the choice of reference genes used for data normalization can influence the conclusions derived from gene expression studies.

## Conclusions

In this study we present a methodology based upon permutation test analysis of SAGE data, to identify reference genes that more stringently satisfy the constancy requirements crucial for accurate normalization between samples utilized in gene expression experiments. Specifically, we have identified reference genes more effective for normalization than the traditional and previously reported housekeeping genes for lung, breast, and brain cancer gene expression profiling. Furthermore, we strongly emphasize that reference genes utilized for expression profiling should be selected in a tissue specific manner. Given that this methodology utilizes sequence-based data, its utility will increase as data generated from new next-generation sequencing platforms accumulate. The usage of more appropriate reference genes will have an impact on the interpretation of existing microarray data as well as expression data generated in future studies, and potentially will shed new insight into the molecular biology of cancer.

## Competing interests

The authors declare that they have no competing interests.

## Authors' contributions

RC analyzed the SAGE and quantitative PCR data to identify the key findings, and wrote the manuscript. KML led the construction of all lung SAGE libraries, contributed to interpretation of data and manuscript editing. LAP performed the quantitative PCR experiments and data interpretation. BPC and CEM provided insight into the statistical analysis as well as manuscript editing. CQZ contributed to the experimental work. THWC contributed to the data analysis. SL isolated the clinical samples, contributed to interpretation of results. CEM, MST, SL, RTN and WLL are the principal investigators on the project. They contributed to the overall design and data interpretation. All authors have read and approved the final manuscript.

## Pre-publication history

The pre-publication history for this paper can be accessed here:

http://www.biomedcentral.com/1755-8794/3/32/prepub

## Supplementary Material

Additional file 1 **Summary of lung SAGE libraries**. Raw tag count summary for lung SAGE libraries used in analysis. Click here for file

Additional file 2 **Summary of breast SAGE libraries**. Raw tag count summary for breast SAGE libraries used in analysis. Click here for file

Additional file 3 **Summary of brain SAGE libraries**. Raw tag count summary for brain SAGE libraries used in analysis. Click here for file

Additional file 4 **Taqman probe IDs of genes assessed by quantitative RT-PCR**. Applied Biosystems Taqman probe IDs for genes assessed by qRT-PCR. Click here for file

Additional file 5 **Probes used for NEPS genes represented on the Affymetrix U133A microarray**. Probes for NEPS genes represented on the Affymetrix U133A microarray. Click here for file

Additional file 6 **Probes used for NEPS genes represented on the Agilent microarray**. Probes used for NEPS genes represented on the Agilent microarray. Click here for file

Additional file 7 **Permutation scores of previously identified and NEPS identified reference genes**. Data used in scatter plot shown in Figure 1. Click here for file

Additional file 8 **Ingenuity Pathway Analysis using genes from the analyses of a NEPS-normalized and unnormalized dataset by Landi et al**. Ingenuity Pathway Analysis using genes from the analyses of a NEPS-normalized and unnormalized dataset by Landi *et al*. Click here for file

Additional file 9 **SAM and pathway analysis of an Agilent lung cancer microarray dataset normalized with and without lung NEPS genes**. *SAM *and pathway analysis of a dataset normalized with and without lung NEPS genes. (A) Number of probes identified as differentially over and underexpressed between cancer and normal using *SAM *on the dataset with and without NEPS normalization. Venn diagram illustrates the overlap in the genes identified as well as those which are different between the two analyses. (B) Canonical pathway analysis using Ingenuity Pathway Analysis. Dark blue bars represent the results from the dataset normalized with NEPS and median normalization and light blue bars represent the results from using median normalization alone. While similar pathways are statistically significant, each pathway is slightly different in the degree of statistical significance. Such differences illustrate the impact of reference gene selection and normalization on differential gene expression analysis. Click here for file

Additional file 10 **Ingenuity Pathway Analysis using genes from the analyses of a NEPS-normalized and unnormalized dataset by Boelens *et al***. Ingenuity Pathway Analysis using genes from the analyses of a NEPS-normalized and unnormalized dataset by Boelens *et al*. Click here for file
