# Supplementary figures and images for: A sequence-based approach to identify reference genes for gene expression analysis
Source: BMC Med Genomics. 2010 Aug 3;3:32. doi: 10.1186/1755-8794-3-32 (PMC2928167; doi:10.1186/1755-8794-3-32)

A

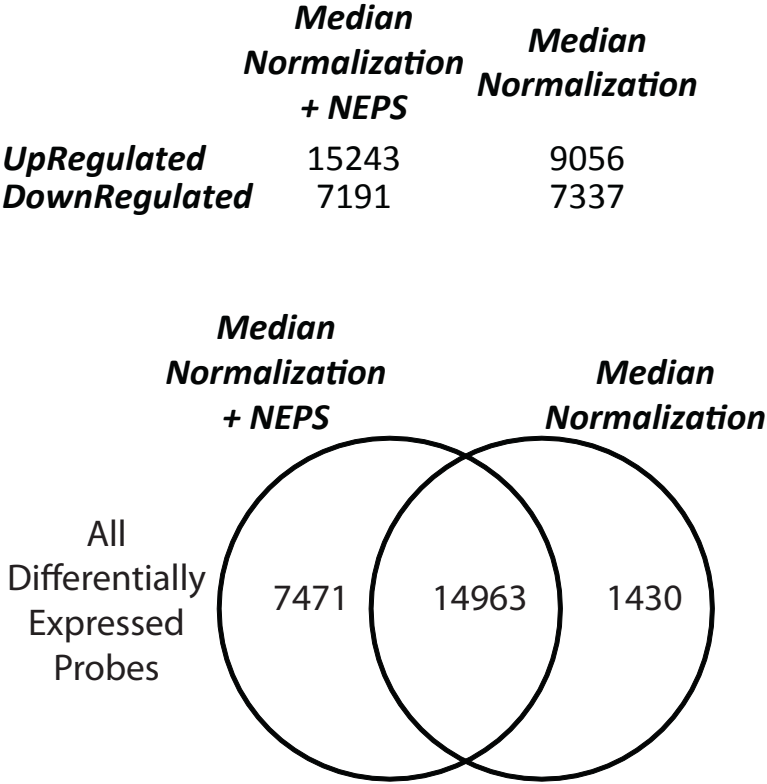

B

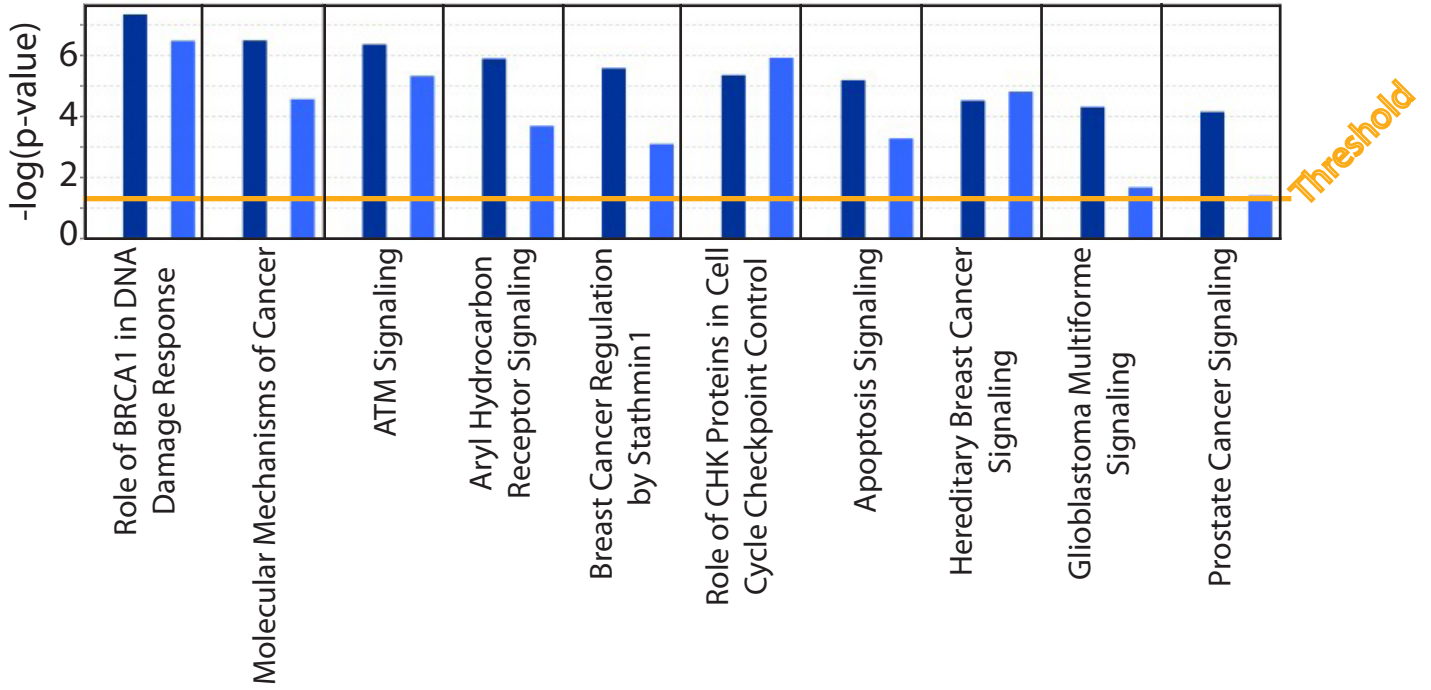

Supplement: Additional file 9 — SAM and pathway analysis of an Agilent lung cancer microarray dataset normalized with and without lung NEPS genes. SAM and pathway analysis of a dataset normalized with and without lung NEPS genes. (A) Number of probes identified as differentially over and underexpressed between cancer and normal using SAM on the dataset with and without NEPS normalization. Venn diagram illustrates the overlap in the genes identified as well as those which are different between the two analyses. (B) Canonical pathway analysis using Ingenuity Pathway Analysis. Dark blue bars represent the results from the dataset normalized with NEPS and median normalization and light blue bars represent the results from using median normalization alone. While similar pathways are statistically significant, each pathway is slightly different in the degree of statistical significance. Such differences illustrate the impact of reference gene selection and normalization on differential gene expression analysis. [file 1755-8794-3-32-S9.PDF]
